# How to Get Better: Taking Notes Mediates the Effect of a Video Tutorial on Number Series

**DOI:** 10.3390/jintelligence9040055

**Published:** 2021-11-18

**Authors:** Benedikt Schneider, Jörn R. Sparfeldt

**Affiliations:** Department of Educational Science, Saarland University, Campus A 5 4, D-66123 Saarbrücken, Germany

**Keywords:** number series, number sequences, video tutorial, notes, mediation

## Abstract

In recent studies, test-score increases have been shown in rule-based intelligence test tasks, such as number series, after watching a corresponding video tutorial. An open question remains regarding the mechanisms involved. Specifically, taking notes to describe the relations between numbers might be linked to test scores, and is hypothesized to mediate the effect of a number series video tutorial on number series test scores. Therefore, an experimental group (EG) watching a number series video tutorial (*n* = 58) was compared with a control group (CG) watching an irrelevant tutorial (*n* = 52) before working on number series items. Results revealed higher number series scores in the EG than the CG (*d* = .48), more items with provided notes in the EG than in the CG (*d* = .41), and substantial correlations between the number of items with notes and the number series sum scores in both groups (EG: *r* = .66; CG: *r* = .75). The effect of the video tutorial on the number series sum score was mediated by the number of items with notes (indirect effect = 3.41, *SE* = 1.74). Theoretical and practical implications as well as future research directions are discussed.

## 1. Introduction

Studies investigating video tutorials showed significant intelligence test-score increases for popular rule-based task types, such as figural matrices or number series of up to large effect sizes (e.g., Schneider et al. 2020, *d* ≥ 1.19; see also Loesche et al. 2015; Schneider and Sparfeldt 2021). Correspondingly, increased test scores might lead to an overestimation of a person’s true cognitive ability, possibly resulting in better chances for selection as well as false selection and admission decisions. The results of these studies imply that watching a video tutorial before an intelligence assessment can be meaningful for the individual test taker. However, the changes in test-taking behavior caused by watching the video tutorial and, thus, the mechanisms responsible for these test-score increases remain largely unknown. We hypothesized that taking notes describing the relations between the numbers of an item represents a generally effective test-taking behavior in number series tasks that might also help clarify the mechanism for test-score increases after watching a video tutorial. Importantly, knowledge about these mechanisms is of relevance to more general questions regarding assessing intelligence and coming to valid interpretations of the test results. In addition to inspecting mean differences in number series test scores after watching a video tutorial, we also investigated whether test takers were taking more notes while solving number series items after watching a number series video tutorial, if these notes were related to higher number series test scores, and if the effect of the video tutorial on number series was mediated by these notes.

### 1.1. Number Series

Number series (or number sequences) are a common task type for assessing numerical reasoning; correspondingly, number series are an integral part of many intelligence test batteries (e.g., Heller and Perleth 2000; Jäger et al. 1997; Kersting et al. 2008; Liepmann et al. 2007; Lohman 2011; Sauerland et al. 2008; Schrank et al. 2014; Weiß 2006; Wonderlic 1992). In each number series item, a sequence of numbers is presented to test takers. These numbers in an item are interconnected through one or more rules repeating periodically in each item. To solve an item, test takers are asked to deduce the rule(s) in the presented sequence and then infer the number that would correctly complement this sequence according to the rule or rules, as indicated by the question mark in the following example.

*Example*: 2 3 6 7 14 15 30?

In this example item, the numbers are interconnected by alternatingly adding one and multiplying by two (i.e., “+1, ×2”). Therefore, the correct number complementing the example sequence is 31.

Concerning the process of solving number series items, four interrelated processing phases are postulated (Holzman et al. 1983; Kotovsky and Simon 1973; see also Holzman et al. 1982; LeFevre and Bisanz 1986; Loe et al. 2018; Verguts et al. 2002). First, test takers create hypotheses about the individual relations of adjacent numbers in the series (relation detection). Second, when test takers discover a number or numbers not in accordance with these hypotheses, the assumption is made that the previously assumed period—that is, a recurring pattern of these relations—is either incomplete, or that a new period is introduced, or that a miscalculation was made (discovery of periodicity). Third, these hypotheses are adapted in regard to the whole number sequence, generating a general solution pattern for the item (completion of pattern description). Finally, this solution pattern is applied to infer the next or missing number in the sequence (extrapolation). In addition to these assumed processing phases, inspecting the descriptions and rules of number series items (e.g., Arendasy and Sommer 2012; Holzman et al. 1983; Loe et al. 2018; Verguts et al. 2002) reveals further commonalities in the structure of such items, which become even more apparent when inspecting the number series items of the previously mentioned published tests. Furthermore, important characteristics of number series items and the solving process of number series items can be summarized efficiently in a parsimonious illustration model (Schneider and Sparfeldt 2021).

### 1.2. Video Tutorials to Increase Test Scores

Test-score increases in intelligence tests in general have been reported for repeated test-taking, coaching, practice, and training (e.g., Hausknecht et al. 2007; Kulik et al. 1984a, 1984b; Scharfen et al. 2018). Typically, such test-score increases reflect test-specific improvements or refined test-taking strategies, and thus do not reflect an enhanced dispositional intelligence (Estrada et al. 2015; Haier 2014; Hayes et al. 2015; Jensen 1998). In consequence, these test-score increases can potentially lead to an overestimation of a test taker’s ability in selection and admission contexts, potentially going hand-in-hand with a mis-fit of the ability of the selected person and the requirements of the selecting institution. For example, such a test taker might be overburdened by the intellectually challenging requirements of a study program or job.

Recent studies showed that watching a short video tutorial in which important characteristics of rule-based intelligence tasks are taught is an effective and efficient approach to increase the corresponding test scores. For example, students of an experimental group who watched a short video tutorial teaching the underlying rules of figural matrices tasks reached higher test scores in an afterwards-administered figural matrices test than students of a control group who watched a task-irrelevant video before the test (e.g., a nutrition video, Schneider et al. 2020; see also Loesche et al. 2015; Krautter et al. 2021; Levacher et al. 2021). Similarly, students of an experimental group who watched a short number series video tutorial based on the mentioned illustration model subsequently solved more number series items than students of a control group who watched a task-irrelevant video tutorial (*d* = .44, Schneider and Sparfeldt 2021). However, the specific mechanisms for the test-score increases caused by watching a video tutorial remain largely unknown. Reasonably, watching a video tutorial illustrating the processes for successfully solving number series items has altered the test-taking behavior to approach the correct solutions more effectively and efficiently.

### 1.3. Taking Notes

**Figure 1:**
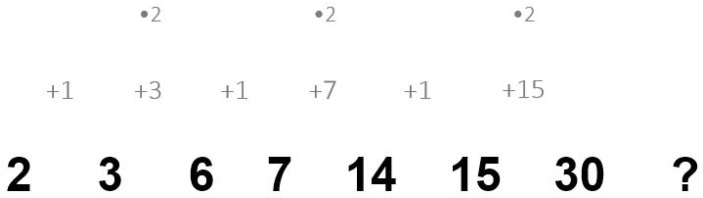
Illustrative example of a number series item with a large number of notated relations.

One potentially viable test-taking behavior for increasing test scores might be to write down relevant information as notes to solve the corresponding item correctly. Test takers can, for example, write down auxiliary calculations, thoughts, or they can mark/highlight difficult parts of an item. Probably, not all forms of notes are useful and, thus, pose as a feasible behavior for solving an item effectively and efficiently. For example, writing down auxiliary calculations and thoughts requires additional time, which might result in a smaller number of solved items due to time constraints and thus ultimately limit its usefulness; marking/highlighting difficult parts of an item (e.g., by underlining numbers that do not yet seem to fit a hypothesized solution pattern) might be practical, but possibly provides little benefit in helping with the solution process (falling in line with research indicating that highlighting is not always useful to improve reading comprehension and learning from texts; e.g., Peterson 1992; Schellings et al. 1996). Ideally, particularly helpful notes would rely on theoretical as well as practical considerations. One useful test-taking behavior that combines these theoretical and practical aspects could be jotting down notes on one or more relations between two numbers as well as the mathematical operation, thereby externalizing elements of the first-mentioned processing phase (i.e., relations detection; e.g., Holzman et al. 1983) and making the detected relations explicit. Such a behavior considers different cognitive functions and goals: (1) identifying and focusing attention on relevant parts of an item while also (2) enabling an easier transfer of this information into working memory (see also Weinstein and Mayer 1986). Furthermore, such written notes reduce working memory requirements (i.e., storage) by externalizing the corresponding information. Additionally, taking useful notes could facilitate subsequent processes of solving number series, such as discovering periodicity (see, e.g., Holzman et al. 1983) and finding the solution pattern—ultimately resulting in an easier inference of the missing number and, thereby, a higher probability of solving the item. Figure 1 illustrates a possible example of thorough and systematic notes with a large number of notated relations. By making the detected relations in such notes explicit, periods are probably easier to discover and the solution pattern becomes more apparent.

A higher number of items reflecting such notes (and thereby a more frequent note-taking behavior) should, thus, be associated with higher scores in a number series test. Furthermore, participants should show this note-taking behavior more frequently after having watched a corresponding number series video tutorial illustrating such notes. Importantly, it seems relevant to keep in mind that some test takers might come up with such a potentially effective and efficient note-taking behavior on their own. As a result, a higher number of items reflecting such notes should also be associated with higher test scores for test takers who did not watch such a video tutorial prior to the assessment. Finally, these considerations also align with theorized explanations for corresponding test-score increases after watching a video tutorial (e.g., increasing the chance to find relevant rules by freeing up cognitive resources; being guided step-by-step through the solution process; Schneider and Sparfeldt 2021). Such a note-taking behavior might thus serve as an intermediary link between a number series video tutorial and the corresponding test-score increases. In other words, the number of items with notes might mediate the effect of watching a number series video tutorial on number series test scores.

### 1.4. The Present Investigation

It has been shown before that watching a number series video tutorial increased the test scores of number series tasks (Schneider and Sparfeldt 2021). However, the mechanisms underlying such test-score increases are largely unknown. These test-score increases should be related to changes in test-taking behavior—possibly, to the notes participants take while working on the items. Therefore, in the present investigation, we examined whether taking notes while working on number series items effected such number series test-score increases. Specifically, we compared the students of an experimental group who watched a number series video tutorial with the students of a control group who watched a task-irrelevant video tutorial regarding the number series test scores, as well as the number of items with notes. As a basis, we expected higher number series test scores for the students of the experimental group than for the students of the control group (as has been shown with these data before, nevertheless forming the foundation for the subsequent analyses regarding the mechanisms of the video tutorial and note taking behavior).^1^

**Hypothesis** **1** **(H1).** *We expected that the students of the experimental group took notes for a higher number of items than the students of the control group.*

**Hypothesis** **2** **(H2).** *Furthermore, we expected substantial and positive correlation coefficients between the amount of number series items with notes and the number series sum scores in both groups.*

**Hypothesis** **3** **(H3).** *Finally, we expected that the effect of watching the number series video tutorial on the number series test scores was mediated by the amount of number series items with notes.*

## 2. Method

### 2.1. Participants and Procedure

The sample under investigation consisted of *N* = 128 teacher-students at a medium-sized German university who attended an obligatory weekly second-year lecture on educational assessment. Based on experiences in the same lecture in former cohorts, regarding the number of participating students, the realized experimental design with the implemented number of groups, and the estimated statistical power regarding the to-be-run statistical tests (e.g., one-tailed *t*-tests (medium effect size), correlations (medium to large effect size)), the aimed sample size of at least *n* = 50 per group was judged to be sufficiently large in order to prevent under-powered statistical tests. Demographic information was assessed about three months before the experiment of this study took place (study year (based on *N =* 115 participants): *M* = 1.74, *SD* = .71; age (based on *N* = 115 participants): *M* = 22.11, *SD* = 4.18; 62.07% female (based on *N* = 116 participants)). Before taking part in the investigation, informed consent was obtained from the participants. Neither number series, in particular, nor intelligence assessment or intelligence, in general, were part of the curriculum before the study took place.

After randomly assigning the participants to the groups^2^, the students of each group were accompanied to a different lecture hall by a trained experimenter. Then, the experiment started with an introductory video sequence. In this introductory video sequence, which was identical in both groups, the students were again welcomed, and the purpose of the investigation was introduced (i.e., information about a widely used task that would help students perform better in future assessments; 33 s). Immediately afterwards, the students of the experimental group (EG; *n* = 63) watched a number series video tutorial (14:25 min; see below), whereas the students of the control group (CG; *n* = 65) watched a figural matrices video tutorial (14:25 min; see below). After the video tutorials had ended, the participants of both groups worked on number series items with German instructions (first, number series items of the I-S-T 2000 R (Liepmann et al. 2007); second, number series items of the WIT-2 (Kersting et al. 2008); third, newly constructed items; see below). To ensure that only those participants were considered in the analyses that worked on the number series items strictly according to the instructions that allowed taking notes, the following analyses were based on those *N* = 110 participants that took at least one note for at least one number series item in every subtest (85.94% of the total sample: EG: *n* = 58; CG: *n* = 52)—ensuring that test takers were aware about this opportunity and were motivated enough to show such a behavior. The study was not preregistered.

### 2.2. Video Tutorials

The video tutorial for the experimental group, which introduced how to solve number series items, and the video tutorial for the control group, which introduced how to solve figural matrices items, were identically designed regarding formal aspects (like duration; i.e., 14:25 min). Additionally, both video tutorials relied on identical instructional principles, such as providing example items, using arrows to illustrate rules, and guiding students step-by-step through the solution process. In the number series video tutorial shown to the students of the experimental group, the processing phases underlying number series (Holzman et al. 1983) were explained, and a notation was introduced in detail illustrating the relationships between the following numbers and the corresponding mathematical operation(s) of a number series item (see Figure 1). In the figural matrices video tutorial shown to the students of the control group, six prominent rules underlying many figural matrices items (e.g., addition and subtraction of symbols) were illustrated and explained (see also Schneider and Sparfeldt 2021).

### 2.3. Instruments and Variables

Students worked on 71 number series items, stemming from three number series subtests. First, we administered the 20 number series items of the subtest “number series” of the I-S-T 2000 R (10 min; Liepmann et al. 2007), a well-established German intelligence test battery. Results reported in the test manual showed convincing evidence regarding psychometric characteristics (e.g., Cronbach’s α = .91) as well as validity (e.g., factorial validity: loading on reasoning −λ = .72). Second, students worked on the 20 number series items of the corresponding number series subtest of the WIT-2 (10 min; Kersting et al. 2008), another well-established German intelligence test battery. In the test manual, convincing evidence regarding, for example, reliability (e.g., Cronbach’s α = .87), as well as validity (e.g., loading on reasoning −λ = .72), is reported. Third, we complemented those two number series subtests by 31 structurally similar and newly constructed number series items in order to reach a higher number of items (16 min; Schneider and Sparfeldt 2021; one further item had to be excluded from the analyses because of a typographical error). All number series items were solvable in accordance with the explanations within the number series video tutorial. Within the instruction, it was indicated that participants could take notes on the answer sheets.

Performance in number series was indicated by the sum score of correctly solved number series items of all 71 items (as well as the three subtest sum scores).

A number series item was considered to include a note when the mathematical relation between two numbers of the number series item was noted at least once in the respective number series item (i.e., an operator as well as a number; e.g., “+3”). Regarding our analyses, we relied on the number of items that included such a note (in total as well as for each of the three number series subtests).

### 2.4. Analyses

Analyses were conducted using SPSS 26 and 27. Supplementary analyses regarding statistical power (a posteriori) were conducted with G*Power v. 3.1 (Faul et al. 2007, 2009). Concerning the mean differences of the number series test scores between the students of both groups, we tested whether the participants of the experimental group solved more items than the students of the control group by running an independent *t*-test with the group as independent variable and the sum score of correctly solved number series items as dependent variable (one tailed; α = .05). In addition, we inspected the effect-size relying on Cohen’s *d* (Cohen 1988; using the pooled standard deviation). Concerning the first hypothesis, which is related to the number of items with notes, we tested whether the participants of the experimental group took notes in more items than the students of the control group by running an independent *t*-test with the group as independent variable and the number of items with notes as dependent variable (one tailed; α = .05; supplemented by Cohen’s *d*). Regarding the second hypothesis, we inspected the Pearson correlations between the number of items with notes and the number series sum score in each group and tested whether the respective coefficients differed statistically significantly from zero (one tailed, α = .05). Concerning the third hypothesis, we ran a mediation analysis (with the program PROCESS v3.5; Hayes 2018) using x = group (predictor; EG = 1, CG = 0), m = number of items with notes (mediator), and y = number series sum score (criterion). Specifically, we inspected the 95% bootstrapping confidence interval of the indirect effect as recommended by Hayes (2018, number of bootstrap samples = 10,000). Additionally, we ran these analyses separately for each of the three number series subtests by using the number of items with notes in each subtest as well as using the corresponding number series subtest score.

## 3. Results

Concerning the analyses related to the number series test scores (see Table 1), the students of the experimental group solved statistically significantly more number series items than the students of the control group regarding the sum score (Cronbach’s α = .94; *t*(108) = 2.52, *p* < .01, *d* = .48, statistical power (a posteriori): 1 − β = .80), as well as each subtest score (I-S-T 2000 R (Cronbach’s α = .86): *t*(108) = 2.40, *p* < .01, *d* = .46, 1 − β = .77; WIT-2 (Cronbach’s α = .86): *t*(108) = 2.54, *p* < .01, *d* = .48, 1 − β = .80; newly constructed (Cronbach’s α = .85): *t*(*df_adjusted_* = 105.56) = 1.98, *p* = .03, *d* = .37, 1 − β = .61). Regarding Hypothesis 1, related to the number of items with notes in both groups, the independent *t*-tests showed that participants in the experimental group took notes when working on more items than participants in the control group (*t*(*df_adjusted_* = 92.67) = 2.09, *p* = .02, *d* = .41, 1 − β = .69). When inspecting the three subtests, a similar result pattern was revealed for the I-S-T 2000 R (*t*(*df_adjusted_* = 92.09) = 2.38, *p* = .01, *d* = .46, 1 − β = .77) and the WIT-2 (*t*(108) = 2.34; *p* = .01, *d* = .45, 1 − β = .76), but not for the newly constructed number series items (*t*(108) = 1.06; *p* = .15, *d* = .20, 1 − β = .27).

Regarding Hypothesis 2, concerning the relations of the amount of number series items with notes and the number series test scores, both variables correlated substantially in the experimental group for the sum score (*r* = .66, *p* < .01, 1 − β > .99) as well as for each subtest (I-S-T 2000 R: *r* = .54, *p* < .01, 1 − β = .99; WIT-2: *r* = .72, *p* < .01, 1 − β > .99; newly constructed items: *r* = .60, *p* < .01, 1 − β > .99). Concerning the control group, the analyses showed a similar result pattern for the sum score (*r* = .75, *p* < .01, 1 − β > .99), as well as for the three subtests (I-S-T 2000 R: *r* = .53, *p* < .01, 1 − β > .99; WIT-2: *r* = .87, *p* < .01, 1 − β > .99; newly constructed items: *r* = .66, *p* < .01, 1 − β > .99). Correlations between all variables are depicted in Table 2.

**Figure 2:**
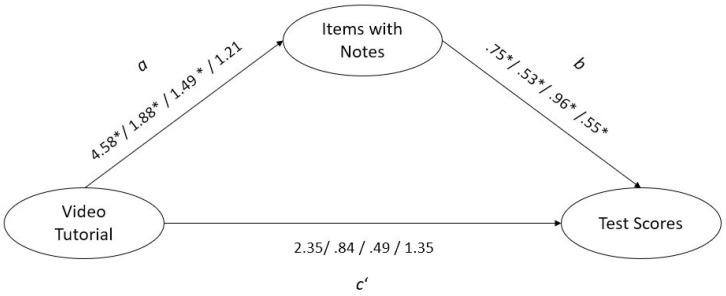
Mediation model. Sum/I-S-T 2000 R/WIT-2/Newly constructed. * *p* < 0.05.

Regarding Hypothesis 3, related to the mediation effects for the sum score (Figure 2), the bootstrap procedure revealed the size of the indirect effect to be 3.41 (*SE* = 1.74); the CI excluded zero (95% CI (.26, 7.06)), indicating that the effect of watching a number series video tutorial on number series test scores was mediated by the number of items with notes. Concerning the three subtests, the results were similar for the I-S-T 2000 R (indirect effect = .99 (*SE* = .47), 95% CI (.18, 2.00)) and the WIT-2 (indirect effect = 1.43 (*SE* = .63), 95% CI (.25, 2.73)). Regarding the newly constructed number series items, however, the confidence interval included zero (indirect effect = .67 (*SE* = .65), 95% CI (–.56, 1.99)). As indicated in Figure 2, all direct effects from the video tutorial to the test scores (c’) failed to be statistically significant.

## 4. Discussion

The present study aimed at clarifying the mechanisms underlying the number series test-score increases caused by watching corresponding video tutorials. Starting from the idea that taking notes while working on number series items might be an effective and efficient behavior playing an important role for solving number series items successfully, we inspected whether the effect of the video tutorial on number series test scores was mediated by the number of items with notes. The results were fourfold: first, the students of the experimental group that had watched a number series video tutorial reached higher number series test scores than the students of the control group that had watched an irrelevant video tutorial (as shown before). Second, the students of the experimental group added notes to a higher number of items than the students of the control group, indicating differences in the test-taking behavior in both groups. Third, the number series test scores correlated substantially (large effect size) with the number of items with notes in both groups. Fourth, taking notes mediated the effect of the video tutorial on number series test scores.

### 4.1. Effects of the Video Tutorial

Regarding number series test scores, the students of the experimental group reached higher number series test scores than the students of the control group, as had been shown before. Thus, we confirmed the number series test score differences as an important prerequisite for meaningfully investigating the corresponding mechanisms of note-taking behavior in students who took at least one note in at least one number series item in each number series subtest. Taking a theoretical perspective, these results imply that showing the processes of how to solve number series items in a video tutorial effectively increases performance scores in number series test items. Taking a more practical perspective, test takers in the real world might search for and watch a corresponding video tutorial—easily available via online search engines and video platforms—while preparing for an upcoming important intelligence assessment to raise their corresponding test scores effectively and efficiently (see also Schneider et al. 2020).

The main focus of our study was related to note-taking behavior while solving number series items, as addressed in the three specified hypotheses. The higher number of items with notes shown by the students of the experimental group than by the students of the control group (Hypothesis 1) indicated that test takers who have watched a corresponding number series video tutorial showed a different test-taking behavior than the students of the control group. Specifically, the students in the experimental group implemented the procedure of taking notes about the relations between the numbers in an item to solve number series items that was illustrated in the video more often than the students in the control group. Thereby, test takers who take such notes more frequently demonstrate a test-taking behavior that reflects the first of the assumed internally organized processes (i.e., relations detection) in manifested behavior. By writing down (one or more) relations between the numbers of a number series item, test takers create an external structure that they can use to distribute their cognitive resources for finding the correct solution effectively and efficiently. Moreover, such an external structure may have different functions that might be fundamentally relevant for all subsequent processes of solving the corresponding item (such as listing different possible mathematical relations between adjacent numbers, storing corresponding relations between adjacent numbers for later use, or scaffolding a systematic process of solving a number series item). Therefore, more research is needed to further clarify the specific function of such a note-taking behavior. However that may be, the number of items with notes seems to be an adequate indicator of an optimized note-taking behavior. If using such an external structure is meaningful for finding the correct solution, a strong link is assumed between the number of items with notes and the number series test scores.

Regarding the correlations addressed in Hypothesis 2, a higher number of items with notes (and therefore a more frequent note-taking behavior) was strongly associated with higher number series test scores in the students of the experimental group watching the number series video tutorial as well as the students of the control group watching the thematically irrelevant tutorial. Therefore, taking notes might indeed be an effective and impactful behavior to increase test scores. However, one should keep in mind that these results are based on correlations, and therefore do not evidence causality (in contrast to the results referring to the effects of the video tutorials on number series test scores and the number of items with notes). Possibly, other influences might be reflected in notes that also contribute to the effect (e.g., a more organized internal processing structure after watching the explanations in the video tutorial that might help improve performance without taking notes). Thus, further research in future studies is required (e.g., by experimentally comparing a group of students allowed to take notes with another group of students prohibited from taking notes). Nevertheless, as the correlations are of substantial magnitude and are similarly high in both groups, the results indicate a plausible as well as considerable general significance of taking notes, as well as the underlying processes for solving number series items correctly.

Regarding the assumed mediation addressed in Hypothesis 3, the results indicate that taking notes mediates the effects of a number series video tutorial on number series test scores. Hence, showing such a note-taking behavior is suggested to be responsible for the associated test-score increases after watching the video tutorial. These results are in line with the hypothesized mediation mechanism. Generally, the processing capacity of human beings is limited. Therefore, test takers have to distribute their cognitive resources efficiently in order to solve an item correctly. By writing down the mathematical operation (as well as the related number) between the numbers of an item, a substantial part of the cognitive capacity otherwise needed to store this information about the relation between adjacent numbers and to keep this information continuously available is freed by externalizing this information into an external storage system (reflecting the first of the assumed processing phases; e.g., Holzman et al. 1983). Presumably, using such an external storage system to keep the corresponding information continuously available allows test takers to allocate more cognitive resources to the subsequent processing phases of the number series item (i.e., finding periods, formulating the solution pattern, and extrapolating the solution to the unknown number). As a result, test takers can distribute their cognitive resources more efficiently, and more cognitive resources are available, for example, to consider the specific interrelations of the mentioned processing phases (e.g., whether relations are repeated or interrupted at regular intervals, indicating the interrelation between relations detection and discovery of periodicity; see Holzman et al. 1983)—ultimately helping them solve an item correctly.

One should keep in mind that the direct effects (c’) of the video tutorial failed to be statistically significant for all dependent variables (number series test scores) in the mediation models. However, concerning the number series sum score and the three number series subtests, the results of the indirect effects were not totally consistent. In contrast to significant mediation effects for the sum score as well as two subtest scores, the mediation analysis revealed no statistically significant mediation effect for the newly constructed items. However, these newly constructed items showed adequate psychometric properties (e.g., Cronbach’s α = .85; substantial correlations with the other subtest scores as seen in Table 2 (.63 ≥ *r* ≥ .76) in similar magnitude as the correlations between the number series of the I-S-T 2000 R and the number series of the WIT-2 as reported in the WIT-2 test manual: *r* = .73; Kersting et al. 2008, p. 127), and thus do not support a hypothesis that insufficient quality of the newly constructed items might be the main reason for these differences in the result pattern. Potentially, for the last set of items, participants’ motivation to use notes was somehow reduced after already working on two other sets of items. Taken together, the result pattern of the mediation models indicated that taking notes plays a crucial role for the effects of watching a video tutorial on number series tasks.

In summary, the results provide valuable insights for a variety of reasons. First, given that video tutorials are easily available and accessible via the internet, the results represent empirical (and experimental) evidence for a potential influence of relevance in a plethora of testing situations. Second, we showed that video tutorials not only influence cognitive processes involved in finding the solution (as had been shown before for, e.g., figural matrices: Loesche et al. 2015; Schneider et al. 2020), but can also stimulate changes in manifested test-taking behavior, going hand-in-hand with these processes and the corresponding test-score increases. Third, in the mediation analyses, we showed that notes are (at least in part) responsible for the corresponding test-score increases, providing evidence that taking notes is indeed a driving factor; this result pattern supports the interpretation of the importance of taking notes that goes beyond a mere association. In conclusion, taking notes is relevant when number series tests are involved. By experimentally demonstrating the relevance of note taking, we implicitly point towards its relevance for test construction. Specifically, further research is needed regarding whether taking notes should be generally encouraged within the test instruction, in consequence even leading up to further questions (e.g., computer-based vs. paper–pencil-based administration and its consequence for taking notes).

### 4.2. Limitations and Future Directions

As for every empirical study, the present study is not without limitations. First, this study focused on a straightforward and general indicator of notes (i.e., a mathematical operation and a number written down in an item to signify at least one relation between the numbers of a number series item). However, test takers might also use other or different types of notes while working on number series items. For example, test takers might use more elaborated notes (e.g., auxiliary calculations), indirect notes (e.g., marking difficult parts or items to work on later during the assessment), or different types of notes related to the subsequent number series processing phases. Future studies might be able to provide a clearer picture about the effectivity and efficiency of different kinds of note-taking behavior by comparing these and other different types of notes, especially related to increased resources regarding other processing phases (e.g., notes indicating periods, notes reflecting the solution pattern). Nevertheless, we highlighted the impact of note taking by investigating an efficient and—by focusing on the first of the processing phases—particularly valuable type of notes in number series tasks. One should keep in mind that we focused on participants taking at least one note in the tests for the analyses. Thereby, we ensured that the results represent test takers being generally aware of the opportunity to take notes as well as being motivated to show such a behavior. Future studies might investigate scenarios where note taking is required, or focus on the potentially very different reasons why participants do not use notes (e.g., lack of motivation, lack of necessity).

Second, as the notes were investigated by inspecting the paper–pencil worksheets after the tests had been completed, an open question remains regarding how precisely these notes are intertwined in the solution process. Theoretically, the notes could have been taken when starting to work on an item (e.g., in the beginning before even inspecting the full sequence), during the solution process (e.g., as a help when the item is more difficult than expected), or even after completing an item (e.g., to double-check whether an intended solution is plausible). Thus, an open question remains regarding if the investigated notes reflect a systematic preparation strategy, a fallback strategy when familiar approaches fail to be expedient, or maybe even a strategy to increase confidence in an established solution. In this context, investigating the efficiency of notes for items of varying individual difficulty might provide additional insight: when working on individually easy items (that could be solved without additional help or notes), using notes might be redundant or even result in time constraints in a test with a limited time period. In contrast, when working on items of medium difficulty for the individual, using notes might be particularly useful by scaffolding a structured as well as effective procedure and by externalizing important information that might provide further cognitive capacities to solve items that would otherwise appear as too difficult. Furthermore, when working on individually too-difficult items representing individually too-intricate problems, using notes might not be sufficient to find the correct solution. Consequently, an inverted U-shaped relationship between the efficacy of notes and (individual) item difficulty is assumed to result (thus falling in line with research on instructional support and strategy use, e.g., Kalyuga et al. 2003; Seufert 2003; see also Weise et al. 2020). Future studies might disentangle these possibilities by presenting the number series tasks in a computerized version, followed by an inspection of corresponding log files about the note taking behavior and the provided solution (and potentially accompanied by additional methods like thinking aloud and/or eye-tracking, thereby combining process data with product data).

Third, one should keep in mind that we specifically allowed test takers to take notes on the same sheets that were used to present the items. Following the administration guidelines and instructions of number series tests, test takers are often prohibited from writing down notes directly on the test sheets (e.g., I-S-T 2000 R; Liepmann et al. 2007). Consequently, the opportunity to take notes above or between the numbers of a number series item is removed, barring an efficient use of such a note-taking behavior. Although it would still be possible for test takers to write down number sequences on a separate sheet and take additional notes, such an approach would be quite time-consuming. As a result, the impact of using notes effectively and efficiently, as is demonstrated in our study, would be reduced in these practical contexts. However, further studies are required to clarify this aspect empirically (e.g., by experimentally varying the opportunity to take notes: allowing notes on the same page vs. allowing notes on a separate sheet vs. not allowing notes at all).

In summary, our study showed that taking specific notes serves as an effective and efficient behavior for increasing test scores in number series tasks. Furthermore, using such an approach can be taught efficiently and illustratively in a number series video tutorial—and is suggested to be responsible for these test-score increases.

## Figures and Tables

**Table 1:** Descriptive statistics for the number of items with notes and the number series scores.

	Experimental Group (*n* = 58)		Control Group (*n* = 52)	
	*M*	*SD*	*M*	*SD*
**Items with notes**				
Sum	57.50	9.48	52.92	12.95
I-S-T 2000 R	16.91	3.39	15.04	4.68
WIT-2	17.10	2.83	15.62	3.81
Newly constructed	23.48	5.41	22.27	6.60
**Number series scores**				
Sum	51.53	12.06	45.77	11.90
I-S-T 2000 R	16.47	3.69	14.63	4.33
WIT-2	14.88	3.65	12.96	4.27
Newly constructed	20.19	6.03	18.17	4.63

**Table 2:** Correlations between all variables in each group.

	1	2	3	4	5	6	7	8
**Experimental Group (*n* = 58)**								
1. Notes: Sum	–	0.69 *	0.87 *	0.86 *	0.66 *	0.57 *	0.63 *	0.58 *
2. Notes: I-S-T 2000 R		–	0.58 *	0.28 *	0.36 *	0.54 *	0.32 *	0.19
3. Notes: WIT-2			–	0.64 *	0.69 *	0.61 *	0.72 *	0.57 *
4. Notes: Newly constructed				–	0.56 *	0.34 *	0.52 *	0.60 *
5. Scores: Sum					–	0.85 *	0.92 *	0.92 *
6. Scores: I-S-T 2000 R						–	0.77 *	0.63 *
7. Scores: WIT-2							–	0.76 *
8. Scores: Newly constructed								–
**Control Group (*n* = 52)**								
1. Notes: Sum	–	0.80 *	0.83 *	0.92 *	0.75 *	0.72 *	0.66 *	0.66 *
2. Notes: I-S-T 2000 R		–	0.51 *	0.57 *	0.43 *	0.53 *	0.34 *	0.29 *
3. Notes: WIT-2			–	0.68 *	0.86 *	0.72 *	0.87 *	0.72 *
4. Notes: Newly constructed				–	0.68 *	0.62 *	0.54 *	0.66 *
5. Scores: Sum					–	0.88 *	0.92 *	0.90 *
6. Scores: I-S-T 2000 R						–	0.74 *	0.65 *
7. Scores: WIT-2							–	0.75 *
8. Scores: Newly constructed								–

Note. * p < 0.05 (two-tailed). Notes = Items with notes. Scores = Number series scores.

## Data Availability

Data is available at https://osf.io/vwq5k/.

## Notes

1 The data and material used in this study were provided by Schneider and Sparfeldt (2021). In the former study, a third group that is irrelevant to the present study was also investigated. While the students of the two groups included in this study were watching the corresponding video tutorial, the students of the not-included third group inspected a small number of number series items by themselves; furthermore, those students were instructed not only to deal with these number series items on their own, but they were also instructed that they could use a dedicated space on the booklet to take notes. Additionally, those students were allowed to switch between a page of the testing booklet with the number series items and another page with the solutions. To summarize, there were substantial qualitative differences regarding note taking realized for this third group and for the two video tutorial groups.

2 Regarding preliminary analyses inspecting whether the realized randomization procedure resulted in sufficiently comparable groups regarding intelligence, we relied on data stemming from a data collection realized about three months before the experiment took place. Based on the sum score of another intelligence test (IST-Screening, Liepmann et al. 2012; consisting of *k* = 20 number series items to assess numerical reasoning, *k* = 20 verbal analogies to assess verbal reasoning, and *k* = 20 figural analogies to assess figural reasoning as well as the sum score indicating an approximation of general intelligence) available for *N* = 99 participants, there were no statistically significantly mean differences between the students of both groups: *t*(df_adjusted_ = 93.39) = .42; *p* = .67.
